# Heterogeneity in functional genetic screens: friend or foe?

**DOI:** 10.3389/fimmu.2023.1162706

**Published:** 2023-06-16

**Authors:** David W. Vredevoogd, Daniel S. Peeper

**Affiliations:** Division of Molecular Oncology and Immunology, Oncode Institute, Netherlands Cancer Institute, Amsterdam, Netherlands

**Keywords:** heterogeneity, CRISPR-Cas9, genetic screens, therapy resistance, TNF

## Abstract

Functional genetic screens to uncover tumor-intrinsic nodes of immune resistance have uncovered numerous mechanisms by which tumors evade our immune system. However, due to technical limitations, tumor heterogeneity is imperfectly captured with many of these analyses. Here, we provide an overview of the nature and sources of heterogeneity that are relevant for tumor-immune interactions. We argue that this heterogeneity may actually contribute to the discovery of novel mechanisms of immune evasion, given a sufficiently large and heterogeneous set of input data. Taking advantage of tumor cell heterogeneity, we provide proof-of-concept analyses of mechanisms of TNF resistance. Thus, consideration of tumor heterogeneity is imperative to increase our understanding of immune resistance mechanisms.

## Introduction

The utility of functional, CRISPR-Cas9 genetic screens in understanding immune resistance mechanisms and, by extension, their value in identifying novel therapeutic targets has become increasingly clear in recent years. Multiple research groups have used such screens to elucidate immunologically active pathways in tumor cells and presented strategies to (therapeutically) exploit them to combat cancer, both *in vitro* (1–11) and *in vivo* (5, 12–15). *In vitro*, such screens have almost invariably been performed with genome-scale libraries in one (or few) tumor cell line(s), whereas *in vivo* screens have been performed using smaller, focused libraries in single tumor cell lines. The reason that screens have largely been limited to single cell lines in publications is a technical one: to ensure maintenance of library complexity (i.e., sufficient replication of each genetic perturbation), and thus fidelity and confidence of the hits identified, a(n extremely) large number of cells need to be used in such screens, making the inclusion of multiple cell lines labor-intensive. Despite this limitation, their success and fidelity were demonstrated by virtue of their identification of common pathways by several groups. By and large they comprise the TNF, IFNγ, antigen presentation and autophagy pathways [reviewed by us (16) and others (17, 18)].

However, these genetic screens do occasionally differ in terms of the exact nodes that they discover within the identified pathways, offering glimpses at potential context-dependent vulnerabilities. This is seen most prominently in one of the few publications in which multiple cell lines were employed (5). Because the screens were performed in the same lab, technical and methodological variation is limited. In those parallel screens, the loss of *TRAF2* was able to sensitize all but one tumor cell line to T cell attack. For this gene in particular, we validated that different tumor cell lines may indeed not all be equally dependent on *TRAF2* for their immune resistance, with some cell lines relying (more) on *BIRC2*, whereas others require inactivation of both genes in order to be sensitized to T cell challenge (1). These observations thus underscore the need to scale up these screens to add to their fidelity and offer insight into the context of identified hits. Because of their limited scale, heterogeneity between tumor cell lines in terms of intrinsic immune resistance mechanisms is currently largely ignored in the design of CRISPR-Cas9 screens, limiting our understanding of immune-resistance mechanisms and preventing us from predicting which cell lines and, by extension, which tumors will respond to specific forms of immunotherapy. In this perspective we will outline sources of tumor heterogeneity, how this may negatively influence CRISPR-Cas9 screens and how to take advantage of those mechanisms in the design of these screens.

## Heterogeneity: nature and causes

Tumor heterogeneity exists in different forms and is caused by multiple processes. Intertumor heterogeneity (i.e., the differences between different tumors), intratumor heterogeneity (i.e., the difference between different tumor cells/clones/populations/regions of the same tumor) and heterogeneity in the tumor micro-environment (i.e., the difference in the anatomical location and non-tumor cell infiltration between [different] tumors and/or metastases) all contribute to the *smorgasbord* we term cancer. These mechanisms of heterogeneity not only co-exist, but frequently also actively influence one another. For example, different metastases of the same tumor in distinct anatomical locations may experience different growth signals and thus display preferential outgrowth of different subpopulations (19–21). In addition, the genetic heterogeneity within tumors can surpass even that between tumors of different individuals (22, 23). Even different single cells within the same tumor can have remarkably different characteristics [reviewed in (24)].

This heterogeneity is manifested through a variety of different mechanisms. They can be summarized in four, central concepts: germline differences, genomic instability, selection by exogenous means and obligate co-dependency of tumor subpopulations. For each of these, clinical evidence illustrates how they can result in tumor heterogeneity. Germline differences are perhaps best characterized within hereditary cancers. For example, hereditary breast cancer cases generally have poorer prognoses than sporadic cases (25). Genomic instability also, a core hallmark of cancer-causing mutations and other genomic aberrations such as genetic duplications or deletions, can lead to inter- and intratumor heterogeneity. This can be driven by, for example, enhanced *APOBEC3* activity in late-stage cancers which promotes the stochastic mutation of the tumor genome (26). Furthermore, non-tumor driven selection, for example through therapy, can result in heterogeneity as tumor subclones with therapy-resistant traits are selected for (27, 28). Lastly, tumors can also evolve to be heterogeneous through the common, co-dependent evolution of different tumor cell subpopulations. In such a symbiotic relationship, one population within the tumor provides growth stimuli to another and, in some cases, this may even be reciprocated (29–31).

## Heterogeneity affects immune sensitivity of tumors

**Figure 1:**
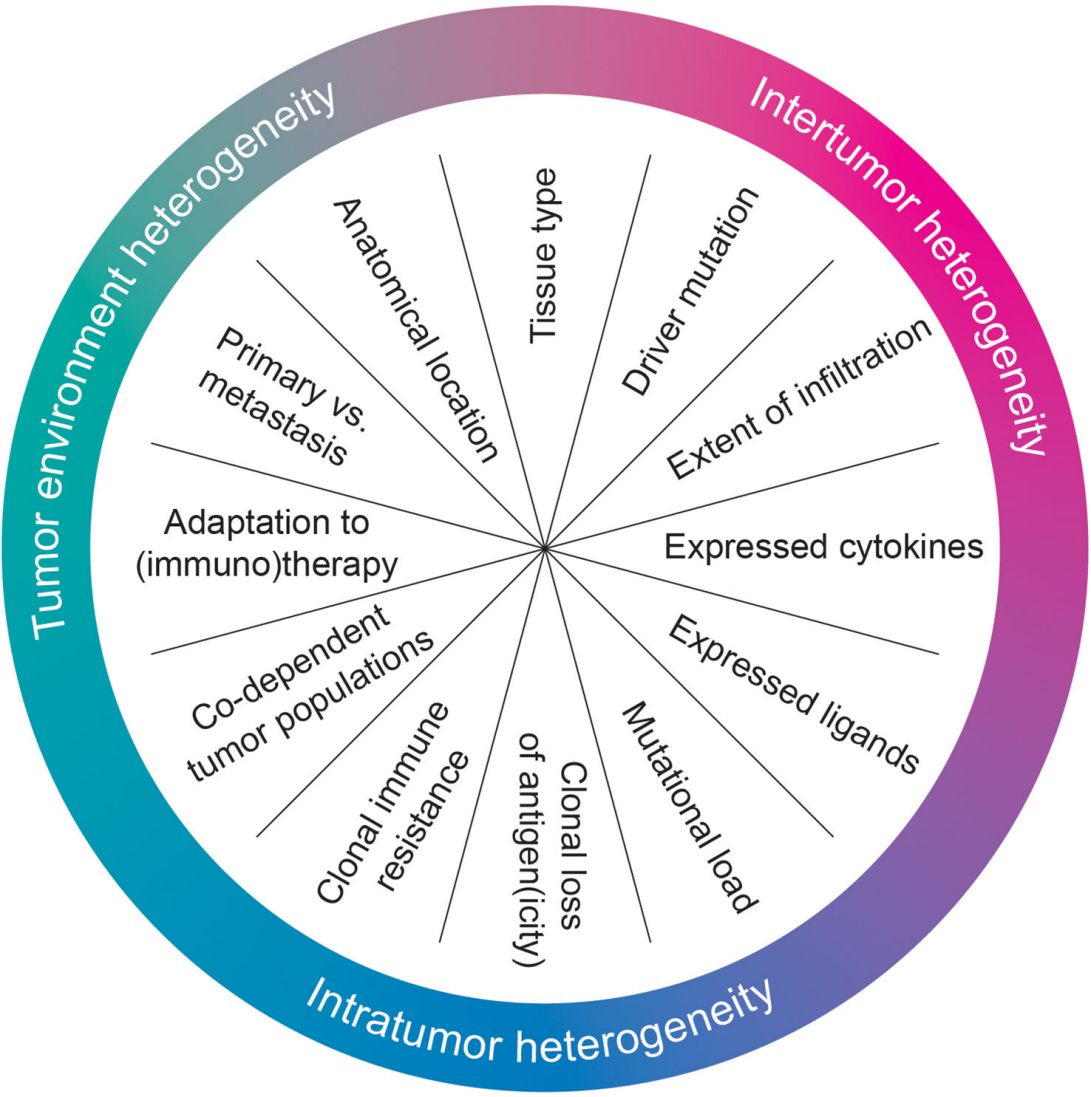
Heterogeneity in immune sensitivity mechanisms.

Heterogeneity can also affect the sensitivity of tumors challenged by multiple different inflammatory cytokines and/or cells of the immune system. This may occur in a general sense, but could also impact specific immune effector pathways. The same general concepts of tumor heterogeneity are involved in these processes (**Figure 1**).

Intertumor heterogeneity is perhaps most evident for the tissue from which a tumor arises. The identity of this tissue in and of itself can already determine immune sensitivity. For example, cancers arising from intrinsically (more) hypoxic tissues, such as melanoma, have heightened expression of cIAP1. These tumors therefore display enhanced resistance against TNF (32). Extending these observations, a recent meta-analysis of tumor-intrinsic determinants of ICB sensitivity identified multiple strong predictors of response for individual tumor types, but those factors fail to predict well in a tumor type-agnostic fashion (33), implying tumor-type specific mechanisms to be at play. Intertumor heterogeneity also manifests through heterogeneity in driver mutations, which can differentially affect the antitumor immune response. An example of this is the generation of an immunosuppressive TME driven by the loss of PTEN (34). KRAS^G12C^ and several p53 mutations, too, alter immune sensitivity (35, 36).

Intertumor heterogeneity can also more broadly influence immune status, being associated with both mutational load (37) and immune infiltrate (38). Each of these may influence which type of immune pressure, and of what strength, a tumor encounters. Another determinant concerns the expression of activating and inhibitory immune ligands, which also differ between tumors and/or tumor types. This heterogeneity in receptor expression may occur upon induction by signals from the TME, such as the differential strength of induction of PD-L1 in different tumors (and tumor cell lines) (39, 40). This phenomenon is particularly of interest as PD-L1, being the main ligand for the inhibitory T cell checkpoint PD-1, is a key target for immune-checkpoint blockade (41–43). Diversity in receptor expression may also be more deeply ingrained, such as the genetically-encoded, patient-specific repertoire of inhibitory receptors for NK cells (immune effector cells that rely on a combined input of activating and inhibitory ligands for their activation) (44–47). This is not only true for cell-surface bound ligands, but equally for tumor cell-derived cytokines or other soluble factors secreted (only) by specific tumors. For example, tumor cell-derived CCL2 indirectly dampens CD8^+^ T cell responses (48) while, additionally, induction of the Wnt/β-catenin signaling pathway leads to T cell exclusion (49).

Intratumor heterogeneity can equally influence immune sensitivity. While some of the above mechanisms may also be evident within a tumor, such as local expression of cytokines and/or immune ligands, other phenomena are also at play. For example, regions within the tumor can lose components of the antigen-presentation machinery, specific T cell antigens or HLA alleles, limiting T cell recognition (50–52). At the same time, such tumor adaptations may (locally) attract otherwise absent immune cells, as was recently shown for Vδ1 and Vδ3 T cells in *B2M ^MUT^* colorectal cancer (53). Additionally, tumor subclones can contain mutations in key immune signaling nodes, even before onset of therapy. They include mutations in *JAK1*, responsible for transmitting IFNγ signals, and in *CASP8*, responsible for the final, decisive step in the apoptotic cascade initiated by TNF (54, 55). Furthermore, different, interdependent subpopulations may contribute to intratumor heterogeneity. In a particularly elegant study, it was demonstrated that IFNγ pathway-mutant tumors are more sensitive to CD8^+^ T cell-mediated eradication due to the loss of protection by IFNγ-induced PD-L1, but become more resistant when intermixed with PD-L1-producing wildtype tumor cells (56). This intratumor heterogeneity is enhanced once (immuno)therapy is administered to the patient tumor, with ample opportunity for selection of escape mutants (52, 57–64).

Lastly, the anatomical location of the tumor may affect immune sensitivity. First, there is a purely technical consideration: the way in which immune sensitivity mechanisms are studied influences how the biology of the pathway manifests. For example, IFNγ has seemingly opposing effects on tumor cell viability *in vitro* and *in vivo*: the cytostatic effects of IFNγ largely inhibit tumor cell growth *in vitro*, whereas *in vivo*, the induction of PD-L1 by IFNγ provides a strong, cytoprotective effect that overcomes those inhibitory effects (16, 65, 66). Additionally, and perhaps obviously, some immune pathways cannot be studied at all *in vitro* because of the use of simplified model systems: either cell types, ligands or cytokines can be missing. The influence of tumor location on heterogeneity has also been demonstrated clinically: different distant metastases may have entirely different TMEs, (neo)antigen burden and immune resistance mechanisms (19, 63, 67, 68). Along these lines, a recent meta-analysis of >2,000 patients showed that genetic alterations in IFNγ signaling components that are present prior to treatment do not necessarily diminish ICB response (69).

## Approach to counteract heterogeneity in CRISPR-Cas9 immune screens

Heterogeneity thus has near limitless influence on the sensitivity of tumors to eradication by the immune system. How can we meaningfully combat, and perhaps even exploit, this heterogeneity in CRISPR-Cas9 screens for tumor-intrinsic, immune sensitivity modifiers? By integrating large amounts of functional screening and *omics* data from many different settings and contexts, one can more precisely annotate tumor cell nodes of immune sensitivity. Specifically, this integration will yield either biomarkers, which mark cell lines in which a particular immune sensitivity node is active, or will generate mechanistic hypotheses that explain why a given node is seemingly inactive in a given cell line. Based on the mechanistic sources of heterogeneity described above, ideally one would derive *omics* and screening data from as many sources as possible. These would include (epi)genomic, transcriptomic and proteomic *omics* data. At the same time, the screening data should be derived from both *in vitro* and *in vivo* screens from as many genetic backgrounds as possible [reviewed in (16)]. Such an undertaking however, would require immense investments of both time and funding.

## Proof-of-concept analyses exploiting cell line-to-cell line heterogeneity

While a comprehensive catalogue of screening data is currently lacking, other domains of research have already embraced the concept of heterogeneity more comprehensively. In fact, in order to find an Achilles’ heel for specific cancers, many cell lines have already been deeply characterized. A multi-decade, multi-national effort, collected within the DepMap database, has screened >1800 cell lines using genome-scale perturbation libraries to identify cancer (type)-specific dependencies. Aside from these functional genetic screens, the cell lines used in these studies have also been extensively characterized, including the collection of RNA, DNA, epigenetic, metabolic and drug-sensitivity metrics (70–72). The use of these databases has allowed investigators to identify novel therapeutic targets in a variety of cancer indications (73–75). An important element lacking from this database then, is an annotation of which genes can be considered immune sensitivity modifiers.

**Figure 2:**
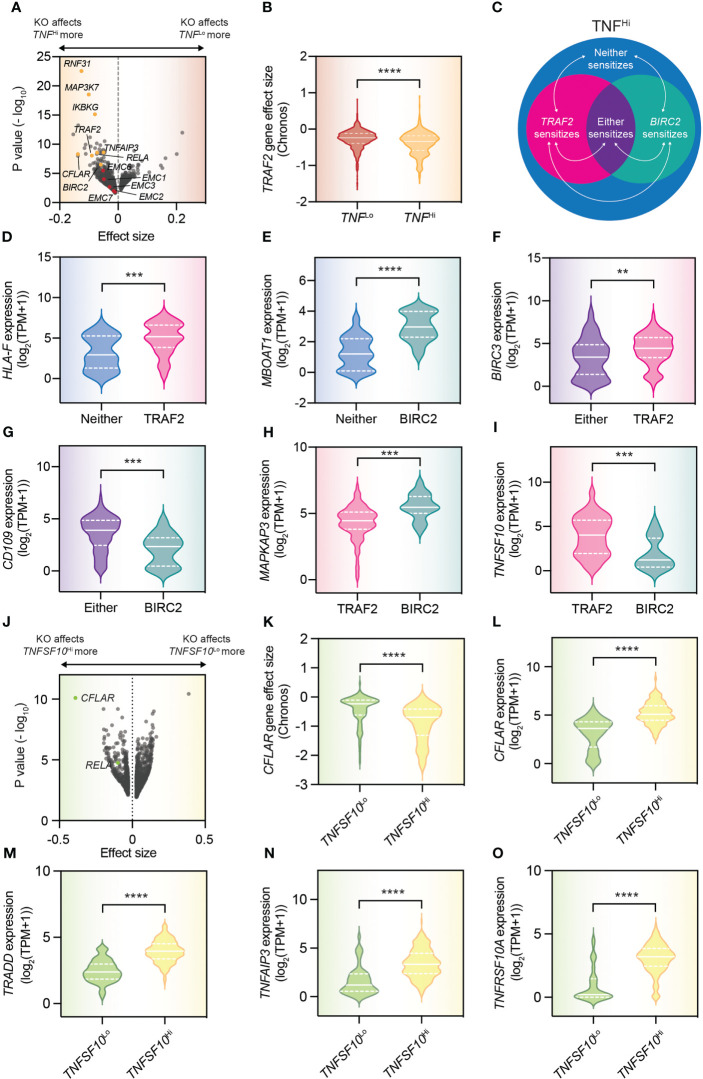
DepMap dependency analyses allow understanding of heterogeneity in immune resistance mechanisms. **(A)** Volcano plot that compares the gene perturbation effects in TNF^Hi^ [i.e., >0.5 log_2_(TPM+1)] and TNF^Lo^ (i.e., 0 read counts for TNF) cell lines. **(B)** Comparison of the effect of *TRAF2* knockout in TNF^Hi^ and TNF^Lo^ cell lines. **(C)** Schematic diagram indicating the populations analyzed in the panels that follow. Only TNF^Hi^ cell lines were used in the analyses. **(D–I)** Violin plots of the expression of indicated genes for the indicated populations (cell lines were deemed sensitive when their CERES score was < -0.3 and insensitive when their CERES score was > -0.1). Statistics were performed by Student t test. The solid white line indicates the population median, with the bottom and top dashed white lines indicating the first and third quartiles, respectively. **(J)** Volcano plot comparing the gene perturbation effects in *TNFSF10* ^Hi^ [i.e., >5 log_2_(TPM+1)] and *TNFSF10* ^Lo^ (i.e., 0 read counts for *TNFSF10*) cell lines. **(K)** Comparison of the effect of *CFLAR* knockout in *TNFSF10* ^Hi^ and *TNFSF10* ^Lo^ cell lines. **(L–O)** Violin plots of the expression of indicated genes for the indicated populations. Statistics were performed by Student t test. The solid white line indicates the population median, with the bottom and top dashed white lines indicating the first and third quartiles respectively. **p < 0.01, ***p < 0.001, ****p < 0.0001.

Interestingly, because of the extent of this database, both in terms of cell line number and cell line characterization, we can perform a proof-of-concept analysis for immune sensitivity modifiers that exploit heterogeneity. Specifically, we can look at modifiers of TNF sensitivity. As more than 300 cell lines in the DepMap produce TNF, we can compare the effects of gene knockouts in these cell lines compared to those that do not produce TNF, to identify factors sensitizing tumor cells to TNF (which, using the excellent portal is trivial to accomplish). By performing this analysis, we could find factors whose ablation reduces viability of TNF^Hi^ cell lines specifically (**Figure 2A**). Indeed, many of those we had already identified and validated ourselves, including *TRAF2*, *BIRC2* (encoding cIAP1) and *RNF31* (**Figures 2A, B**) (1, 2). However, with such an approach we could identify also novel, potential TNF sensitivity modifiers, such as the EMC family of genes which, though currently not yet validated, we also identified in our meta-analysis of immune sensitivity screens (**Figure 2A**) (16).

Having established the fidelity of this approach, we could continue by also taking advantage of the size and heterogeneity of the particular database used. In our previous work, we have identified a differential reliance on *TRAF2* and *BIRC2* to establish resistance to TNF in different tumor cell lines. While it had been difficult to fully comprehend this differential sensitivity before, given that TRAF2 and cIAP1 are thought to signal in a linear fashion, we could now make transcriptomic comparisons between TNF^Hi^ cell lines in which both *TRAF2* and *BIRC2* sensitize, those in which solely *BIRC2* knockout sensitizes, those in which solely *TRAF2* knockout sensitizes, or those cell lines in which neither the loss of *TRAF2* nor the loss of *BIRC2* reduces the viability of the affected cell line (**Figure 2C**). These analyses can yield biomarkers of specific populations (**Figures 2D–J**). For example, high *HLA-F* expression marks populations that will respond solely to *TRAF2* inhibition (**Figure 2D**). These analyses can also provide mechanistic insight. For example, the observation that *BIRC3* expression is higher in cell lines that respond solely to the loss of *TRAF2* compared to those that respond to either *TRAF2* or *BIRC2* loss, implies that this protein compensates for the loss of its paralog *BIRC2* (**Figure 2F**).

As a second proof-of-concept for discovery of immune sensitivity modifiers that exploit heterogeneity using the DepMap, we performed a similar analysis for cells producing TNF-related apoptosis-inducing ligand (TRAIL, encoded by the gene *TNFSF10*; **Figure 2J**). Here, we identified the loss of both *CFLAR* and *RELA* to specifically sensitize those cells capable of producing TRAIL, in line with published literature (**Figures 2J, K**) (76, 77). Using the transcriptomic data of those same cell lines, we may even begin to speculate as to how these cells are capable of surviving in the presence of TRAIL. These cells seemingly induce transcription of genes that protect against TRAIL-induced cell death, including the aforementioned *CFLAR*, but also *TRADD* and *TNFAIP3* (**Figures 2L–N**) (76–78). In doing so, they may gain a previously described proliferative advantage of TRAIL signaling (78), which may explain their higher level of expression of the TRAIL receptor, *TNFRSF10A* (**Figure 2O**), but this prediction awaits functional validation.

**Figure 3:**
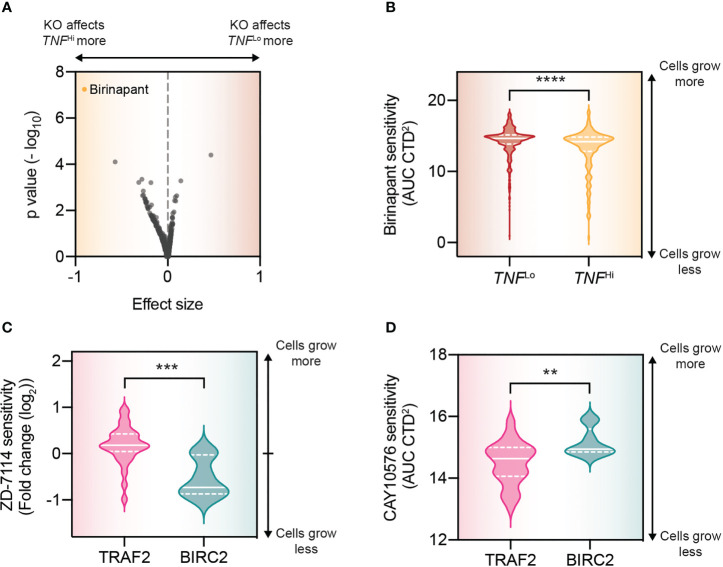
DepMap drug analyses allow for the potential exploitation of heterogeneity in immune resistance mechanisms. **(A)** Volcano plot that compares the drug treatment effects in TNF^Hi^ (i.e., >0.5 log_2_(TPM+1)) and TNF^Lo^ (i.e., 0 read counts for TNF) cell lines. **(B)** Comparison of the effect of birinapant in TNF^Hi^ and TNF^Lo^ cell lines. **(C)** Violin plots of the drug effects of ZD-7114 for the indicated populations. Statistics were performed by Student t test. The solid white line indicates the population median, with the bottom and top dashed white lines indicating the first and third quartiles respectively. **(D)** As **(C)**, but for CAY10576. **p < 0.01, ***p < 0.001, **** p < 0.0001..

Beyond these transcriptomic comparisons, we can exploit the DepMap to find ways of targeting these specific tumor cell subpopulations. Again, to probe the fidelity of such an approach, we compared the drug sensitivity between the TNF^Lo^ and TNF^Hi^ cell lines in the DepMap. With this analysis, we could, at least in part, recapitulate the genetic analysis, identifying birinapant, an inhibitor of cIAP1 to sensitize TNF^Hi^ cell lines more than TNF^Lo^ cell lines (**Figures 3A, B**). We validated this therapeutic approach previously in conditions of high concentrations of TNF (i.e., T cell attack) (1). Using the drug sensitivity database, we could also identify specific inhibitors for the cell lines differentially dependent on *TRAF2* and *BIRC2* for their resistance against TNF. For those cell lines that particularly depend on *BIRC2* we found that ZD-7114, a β3-adrenoceptor agonist, is a potential pharmaceutical strategy (**Figure 3C**). In cell lines that depend on *TRAF2*, we could find a specific sensitivity to CAY10576, an IKKϵ inhibitor (**Figure 3D**). IKKϵ is known interact with TRAF2, and its identification may thus have a clear mechanistic basis (79).

## Considerations for the future

While the above analyses show the promise of integrating heterogeneity in target discovery, they are preliminary and marred by assumptions (e.g., can we realistically assume that TNF-producing cells are a good model for cell experiencing T cell-derived TNF? Can we assume that protein levels of TNF scale linearly with TNF mRNA expression)? Therefore, and as mentioned above, the true complexity of tumor-immune interactions, and forms and mechanisms of heterogeneity at play require more data to be integrated in these models. Firstly, and perhaps most easy to accomplish, the field should invest in performing more tumor : T cell screens, to complement those that have already been reported in key publications in the recent past (1, 3–7, 12–16, 18, 75, 80). These screens, combined with deep characterization as performed for the DepMap, should result in a more granular understanding of genotype – phenotype interactions, as demonstrated here with our proof-of-concept analyses (**Figure 2**). An analogous approach was already taken for NK sensitivity (75). Obviously, such screens only scratch the surface of the different types of heterogeneity outlined above. One could imagine that with time, and significant investment, the screens can be performed in parallel in a large number of settings. For example, they can be performed with different (e.g., NK cells, as was done in (75), or ‘exhausted’ vs. polyfunctional T cells), or more complex co-culture systems (e.g., tumor : T cell : NK cell combinations), more environmental perturbations (e.g., nutrient starvation, hypoxia, highly acidic conditions), in *in vivo* mouse models (as in (5, 12, 13), in isogenic tumor cell lines with specific alterations [as was done in (5)] or even in combination with specific therapeutics (e.g. anti-CTLA-4 or anti-PD-1). Ultimately, such genetic screens will improve our understanding of important immune resistance mechanisms, aiming to have as many patients as possible benefit from (personalized) immunotherapy.

## Author contributions

DV performed the analyses. DV and DP wrote the manuscript. All authors contributed to the article and approved the submitted version.
